# Hospital Meetings

**Published:** 1895-12-07

**Authors:** 


### HOSPITAL MEETINGS.

### THE MIDDLESEX HOSPITAL.—QUARTERLY COURT.

At the quarterly general court of governors of the Middlesex Hospital, held on November 28th, a satisfactory report was given of the progress being made in the construction of the new convalescent home at Clacton-on Sea. The interior fittings of the main building are being proceeded with, and the out-buildings steadily advancing. A sub-committee has been appointed, and a meeting held to consider plans for the new cancer wing, and the architect instructed to prepare draft designs for a building to include three wards to accommodate 30 female patients, isolation wards, and requisite provision for the nursing and domestic staff. The new wing will be approached from the hospital by means of a subway. A further appeal for funds towards the erection of this much-needed addition has been issued; the cost was originally estimated at £12,000, the contributions already received amounting to upwards of £9,250, including a donation of £100 from Lady Cranshaw during the last quarter. The Board having learnt that under the will of the late Mr. F. O. T. Delmar the income of a sum of £100,000 has been bequeathed to trustees for distribution among certain charitable institutions, prompt application was made to them, representing the claims of the new cancer wards to participate in the benefits of the bequest. The decision of the trustees has not yet been made known, but it is much to be hoped that it may be favourable. The advantage of having such special wards in connection with the large general hospitals, instead of as separate establishments, is manifest from many points of view, and the need of more beds for cancer cases is so pressing that there should be no difficulty in obtaining the funds necessary for the speedy completion of the new wing. That the Middlesex Hospital is well to the fore with necessary improvements is shown by the fact that the last quarter has seen the provision of a new steam cooking apparatus for the nurses' home kitchen, and a new hot-water service to the nurses' baths. The vacancy made on the hospital board by the death of Mr. John Bell Sedgwick, late deputy chairman, has been filled by the election of his nephew, Lieutenant-Colonel T. Lambert Mears, while Sir Arthur Townley Watson has been appointed in the place of Colonel Needham.

